# Bone mineral density as an individual prognostic biomarker in NSCLC patients treated with immune checkpoint inhibitors

**DOI:** 10.3389/fimmu.2024.1332303

**Published:** 2024-04-18

**Authors:** Jie Lou, Bingxin Gong, Yi Li, Yusheng Guo, Lin Li, Jing Wang, Weiwei Liu, Ziang You, Hongyong Zhang, Feng Pan, Bo Liang, Lian Yang, Guofeng Zhou

**Affiliations:** ^1^ Department of Ultrasound Medicine, Union Hospital, Tongji Medical College, Huazhong University of Science and Technology, Wuhan, China; ^2^ Hubei Key Laboratory of Molecular Imaging, Wuhan, China; ^3^ Department of Radiology, Union Hospital, Tongji Medical College, Huazhong University of Science and Technology, Wuhan, China; ^4^ Department of Hematology, Union Hospital, Tongji Medical College, Huazhong University of Science and Technology, Wuhan, China

**Keywords:** bone mineral density, osteoporosis, immunotherapy, immune checkpoint inhibitors, non-small cell lung cancer, prognosis

## Abstract

**Background:**

Immune checkpoint inhibitors (ICIs) have left a deep impression in the treatment of non-small cell lung cancer (NSCLC), however, not all patients benefit from it. The purpose of this study was to investigate the prognostic value of baseline bone mineral density (BMD) derived from chest computed tomography (CT) scans in NSCLC patients treated with ICIs.

**Methods:**

This study included patients with advanced NSCLC who underwent ICI treatment at the Wuhan Union Hospital from March 2020 to October 2022. Baseline BMD was evaluated at non-contrast chest CT at the level of first lumbar vertebra. Patients were divided into BMD-lower group and BMD-higher group according to the optimal cutoff value calculated by X-tile software. Baseline characteristics of the two groups were compared and variables between the two groups were balanced by propensity score matching (PSM) analysis. We calculated the objective response rate (ORR) and disease control rate (DCR) of the two groups and analyzed overall survival (OS) and progression-free survival (PFS) using BMD and other clinical indexes through Cox regression models and Kaplan-Meier survival curves.

**Results:**

A total of 479 patients were included in this study, and all patients were divided into BMD-lower group (n=270) and BMD-higher group (n=209). After PSM analysis, each group consisted of 150 patients. ORR (43.3% vs. 43.5% before PSM, *P* = 0.964; 44.7% vs. 44.7% after PSM, *P* = 1.000) and DCR (91.1% vs. 94.3% before PSM, *P* = 0.195; 93.3% vs. 96.7% after PSM, *P* =0.190) were similar in two groups. There was no statistically significant relationship between BMD degree and PFS before (16.0 months vs. 18.0 months, *P* = 0.067) and after PSM analysis (17.0 months vs. 19.0 months, *P* = 0.095). However, lower BMD was associated with shorter OS both before (20.5 months vs. 23.0 months, *P*< 0.001) and after PSM analysis (20.0 months vs. 23.0 months, *P* = 0.008).

**Conclusion:**

Lower baseline BMD is associated with worse clinical outcomes in NSCLC patients treated with ICIs. As a reliable and easily obtained individual prognostic biomarker, BMD can become a routine detection indicator before immunotherapy.

## Introduction

Lung cancer is the leading cause of cancer death worldwide and non-small cell lung cancer (NSCLC) accounts for approximately 85% of all lung cancers (1). Unprecedented advances have been made in the treatment of lung cancer, such as new targeted therapies and immunotherapies (2). Immune Checkpoint Inhibitors (ICIs), including anti-programmed cell death protein 1 (PD-1), anti-programmed death-ligand 1 (PD-L1), and anti-cytotoxic T-lymphocyte-associated protein 4 (CTLA-4), could block inhibitory signals of T cell activation to promote anti-tumor immune response (3). ICIs have greatly prolonged survival for patients with advanced or metastatic NSCLC (4). However, a subset of patients do not derive clinical benefit and may even develop disease progression with ICIs due to systemic factors (5). Therefore, there is an urgent need for reliable predictive biomarkers to identify patients who are suitable for immune checkpoint therapy.

As a chronic wasting disease, malignant tumors cause the prevalence of osteopenia and sarcopenia to be significantly higher than that in people of the same age (6, 7). As a result of malnutrition, these protracted musculoskeletal disorders negatively influence the quality of life ultimately resulting in a poor prognosis. Notably, a number of studies have reported that sarcopenia was significantly associated with increased mortality in patients with NSCLC (8, 9), however, the association between osteopenia and the prognosis of NSCLC has been less frequently reported, especially among patients receiving immunotherapy. T-score evaluated by dual-energy X-ray absorptiometry (DXA) was the gold standard for osteoporosis diagnosis (10). Currently, computed tomography (CT)-derived bone mineral density (BMD) was reported to be correlated with T-score and has been widely used to evaluate preoperative osteopenia in patients with digestive tract cancers (11). Patients with lung cancer routinely receive chest CT scans, and BMD can be obtained non-invasively through measuring the HU value at the level of the first lumbar vertebra.

To the best of our knowledge, there are no studies reporting the impact of baseline BMD on the efficacy of immunotherapy and clinical prognosis in patients with NSCLC. Therefore, we evaluated the objective response rate (ORR) and disease control rate (DCR) after the treatment of ICIs in NSCLC patients with low baseline BMD and high baseline BMD and tried to analyze overall survival (OS) and progression-free survival (PFS) using BMD and other clinical indexes through Cox regression models and Kaplan-Meier survival curves.

## Materials and methods

The local ethics committee and the institutional review board of the Tongji Medical College have approved this retrospective cohort study (Institutional Review Board No. S054), and they waived the requirement for informed consent. Clinical data were analyzed retrospectively and anonymously.

### Study design and patients selection

Consecutive advanced NSCLC patients treated with ICIs between March 2020 to October 2022 at Wuhan Union Hospital were reviewed retrospectively. The diagnosis of NSCLC was based on radiological imaging, medical history, and/or lung biopsy.

Inclusion criteria were as follows: (1) Patients diagnosed with advanced NSCLC according to the NCCN Clinical Practice Guidelines in Oncology: Non-Small Cell Lung Cancer (2); (2) Patients older than 18 years; (3) The follow-up duration was more than 12 months; (4) Performing non-contrast chest CT before initial ICIs; (5) Patient received ICI treatment for the first time and for more than 4 cycles. Exclusion criteria were as follows: (1) Patients who did not undergo baseline CT; (2) Patients combined with other malignant tumors; (3) Patients with incomplete clinical information.

### Procedures

Covariates of interest were collected retrospectively, including patient demographics (age, sex, body mass index, ECOG status, diabetes, hypertension, smoking, hyperlipidemia, COPD), biochemical data (alkaline phosphatase, lactic dehydrogenase, Ca, blood urea nitrogen, hemoglobin, albumin-globulin ratio, neutrophil to lymphocyte ratio, platelet to lymphocyte ratio), tumor-related information (pathological types, stages) and further disease specific information (type of ICIs, prior radiation therapy, occurrence of vertebral bone metastasis, corticosteroid application and osteopenia treatment). All baseline data are derived from the first admission and discharge medical records of patients.

### Bone mineral density measurement and assessment

**Figure 1:**
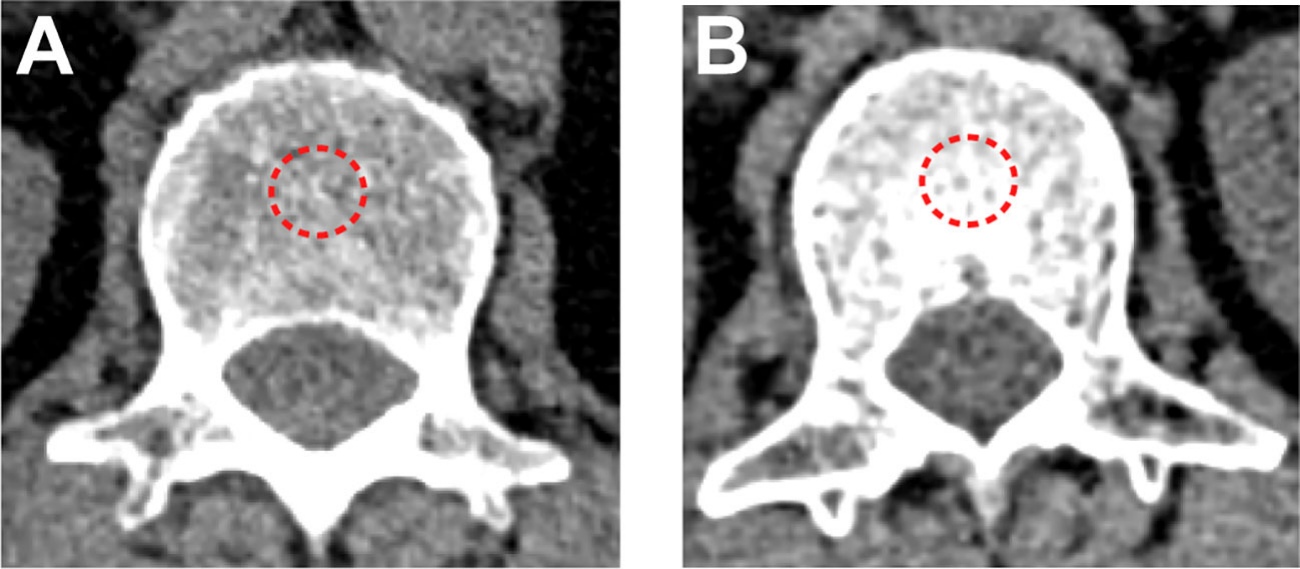
BMD measured by non-contrast chest CT scan. **(A)** A patient in the BMD-lower group (BMD=83 HU). **(B)** A patient in the BMD-higher group (BMD=190 HU). The red dotted line represents the outlined regions of interest.


The CT examinations were performed on the 128-section CT scanner (SIEMENS SOMATOM Definition AS+, Siemens Healthcare Erlangen, Germany) using the same parameters. Tube voltage: 120kVp. Tube current: automatically adjusted. Reconstruction method: standard soft convolution kernel. Slice thickness: 1 mm. Slice interval: 1 mm. Two independent radiologists (L.B. and P.F. with 26 and 15 years of thoracic imaging experience, respectively) analyzed images and calculated the BMD independently on the Phillips Intelli Space Portal workstation (version 10.1, Best, the Netherlands), blinded to the clinical data. The average BMD from two independent radiologists was calculated for subsequent analysis and any disagreements were resolved by consensus. BMD was calculated as the average pixel density (HU) within a circle in the mid vertebral core at the bottom of the first lumbar vertebra on non-contrast chest CT (**Figure 1**). Draw three regions of interest (ROIs) repeatedly and average them to reduce errors. If the difference in HU values among them is greater than 30, another observer would repeat the drawing and calculation. Evaluations were repeated using the same method after two weeks, and intraobserver and interobserver agreements were 0.94 (95% CI, 0.89 to 0.97) and 0.90 (95% CI, 0.84 to 0.93), respectively. Using the X-tile software (version 3.6.1) to obtain the optimal cutoff value, all patients were divided into the BMD-lower group and BMD-higher group. To demonstrate osteopenia, this study also measured BMD at the tenth thoracic vertebra and performed correlation analysis between two BMD measurements from the first lumbar vertebra and the tenth thoracic vertebra. Age-adjusted standard BMD was calculated by the following formulae (12):

BMD (HU) for men=308.82−2.49×Age in years

BMD (HU) for women=311.84−2.41×Age in years

### Definition and evaluation of data

All patients underwent follow-up until October 2023. Follow-up chest CT was compared to the baseline imaging to determine the time of PFS and the ratio of ORR and DCR between the two groups based on the Response Evaluation Criteria in Solid Tumors, version 1.1 (13). ORR and DCR were calculated based on the number of complete response (CR), partial response (PR), and stable disease (SD). PFS was defined as the time elapsed between initial ICI treatment and the onset of tumor progression or patient death. OS was defined as the period from the initial ICI treatment to the last follow-up or patient death.

In addition, we calculated the incidences of skeletal-related events (SRE) in the BMD-lower group and BMD-higher group. SRE include pathologic fracture, need for surgery/radiation therapy to bone and spinal cord compression (14, 15).

### Statistical analysis

The mean and standard deviation of continuous variables were compared using the paired or independent student’s t-test, and the percentages of discrete variables were calculated using the Chi-square test. Correlation was performed using Pearson’s correlation analysis and Spearman’s correlation analysis. The cut-off values (BMD-related indexes) were determined using the X-tile software (Yale University School of Medicine, New Haven, Connecticut, USA). This software provided a comprehensive approach to dividing a cohort into low-level and high-level marker expressions based on survival curves (16). Propensity score matching (PSM) analysis was performed with a caliper value of 0.05 to reduce patient selection bias and to balance the variables between the BMD-lower and BMD-higher groups. One-to-one matching based on baseline characteristics of patients. The Kaplan-Meier method and the log-rank test were used to compare the differences in PFS, and OS in two groups. In the Cox regression analysis, variables with a univariate *P* value less than 0.1 were included in the multivariable Cox regression model. PFS and OS hazard ratios for each subgroup were calculated using unstratified univariate Cox models and presented as forest plots. All the tests were two-tailed; a *P*-value of less than 0.05 denoted statistical significance. All analyses were performed with SPSS version 26.0 software (IBM, Armonk, NY, USA), and R version 4.3.0 (R Foundation).

## Results

### Patient characteristics

This study included 479 patients with advanced NSCLC (270 in the BMD-lower group and 209 in the BMD-higher group), and all of these underwent the treatment of ICIs. **Table 1** shows the baseline demographic and clinical characteristics of patients before and after PSM analysis. Compared with the BMD-higher group, the BMD-lower group had a higher proportion of patients older than 65 years old and a higher prevalence of hypertension. In addition, the BMD-lower group had a higher proportion of patients with ECOG ≥1. These differences were reduced after PSM analysis and reached balance. After PSM analysis, both the BMD-lower group and the BMD-higher group consist of 150 patients (**Table 1**). Baseline characteristics of patients excluded by PSM are shown in **Supplementary Table 1**. Histogram of propensity scores showed a closer distribution of propensity scores between the BMD-lower and BMD-higher groups after matching (**Supplementary Figures 1A, B**).

**Table 1:** Baseline characteristics of patients before and after PSM analysis.

Characteristics	Before PSM			After PSM		
	BMD-lower	BMD-higher	*P* value	BMD-lower	BMD-higher	*P* value
Patients, n	270	209		150	150	
Sex, n (%)			0.107			0.854
Male	231 (85.6%)	189 (90.4%)		133 (88.7%)	134 (89.3%)	
Female	39 (14.4%)	20 (9.6%)		17 (11.3%)	16 (10.7%)	
Age, n (%)			<0.001			0.401
<65	132 (48.9%)	158 (75.6%)		92 (61.3%)	99 (66.0%)	
≥65	138 (51.1%)	51 (24.4%)		58 (38.7%)	51 (34.0%)	
Body mass index, n (%)			0.379			0.667
<Median	223 (82.6%)	166 (79.4%)		118 (78.7%)	121 (80.7%)	
≥Median	47 (17.4%)	43 (20.6%)		32 (21.3%)	29 (19.3%)	
ECOG status, n (%)			<0.001			0.627
0	151 (55.9%)	149 (71.3%)		96 (64.0%)	100 (66.7%)	
≥1	119 (44.1%)	60 (28.7%)		54 (36.0%)	50 (33.3%)	
Pathological types, n (%)			0.307			0.888
Squamous carcinoma	139 (51.5%)	93 (44.5%)		79 (52.7%)	78 (52.0%)	
Adenocarcinoma	115 (42.6%)	103 (49.3%)		63 (42.0%)	62 (41.3%)	
Other	16 (5.9%)	13 (6.2%)		8 (5.3%)	10 (6.7%)	
Stages, n (%)			0.924			0.535
Stage III	76 (28.1%)	58 (27.8%)		50 (33.3%)	45 (30.0%)	
Stage IV	194 (71.9%)	151 (72.2%)		100 (66.7%)	105 (70.0%)	
Type of ICIs, n (%)			0.652			0.531
PD-1	251 (93.0%)	192 (91.9%)		136 (90.7%)	139 (92.7%)	
PD-L1	19 (7.0%)	17 (8.1%)		14 (9.3%)	11 (7.3%)	
Smoking, n (%)			0.411			0.815
Yes	151 (55.9%)	109 (52.2%)		89 (59.3%)	87 (58.0%)	
No	119 (44.1%)	100 (47.8%)		61 (40.7%)	63 (42.0%)	
Diabetes, n (%)			0.606			0.395
Yes	27 (10.0%)	18 (8.6%)		10 (6.7%)	14 (9.3%)	
No	243 (90.0%)	191 (91.4%)		140 (93.3%)	136 (90.7%)	
Hypertension, n (%)			0.011			0.899
Yes	98 (36.3%)	53 (25.4%)		45 (30.0%)	44 (29.3%)	
No	172 (63.7%)	156 (74.6%)		105 (70.0%)	106 (70.7%)	
Hyperlipidemia, n (%)			0.549			1.000
Yes	87 (32.2%)	62 (29.7%)		49 (32.7%)	49 (32.7%)	
No	183 (67.8%)	147 (70.3%)		101 (67.3%)	101 (67.3%)	
COPD, n (%)			0.405			0.274
Yes	28 (10.4%)	17 (8.1%)		20 (13.3%)	14 (9.3%)	
No	242 (89.6%)	192 (91.9%)		130 (86.7%)	136 (90.7%)	
Alkaline phosphatase, mean (SD)	97.7 (48.0)	106.1 (66.4)	0.121	99.0 (48.0)	99.2 (51.6)	0.979
Lactic dehydrogenase, mean (SD)	228.0 (103.7)	245.6 (120.7)	0.087	227.9 (116.9)	233.7 (103.5)	0.645
Ca, mean (SD)	2.2 (0.1)	2.2 (0.2)	0.965	2.2 (0.1)	2.2 (0.1)	0.642
Blood urea nitrogen, mean (SD)	5.4 (1.8)	5.3 (1.7)	0.377	5.5 (1.9)	5.4 (1.8)	0.868
Hemoglobin, mean (SD)	123.0 (16.6)	124.9 (15.7)	0.209	124.1 (15.9)	124.9 (16.3)	0.660
A/G ratio, mean (SD)	1.5 (1.3)	1.4 (0.3)	0.400	1.4 (0.3)	1.4 (0.3)	0.603
NLR, n (%)			0.624			0.222
≤2	42 (15.6%)	36 (17.2%)		30 (20.0%)	22 (14.7%)	
>2	228 (84.4%)	173 (82.8%)		120 (80.0%)	128 (85.3%)	
PLR, n (%)			0.176			0.633
≤150	104 (38.5%)	68 (32.5%)		58 (38.7%)	54 (36.0%)	
>150	166 (61.5%)	141 (67.5%)		92 (61.3%)	96 (64.0%)	
Prior radiation therapy, n (%)			<0.001			1.000
Yes	30 (11.1%)	2 (1.0%)		3 (2.0%)	2 (1.3%)	
No	240 (88.9%)	207 (99.0%)		147 (98.0%)	148 (98.7%)	
Vertebral bone metastasis, n (%)			0.008			1.000
Yes	68 (25.2%)	32 (15.3%)		22 (14.7%)	22 (14.7%)	
No	202 (74.8%)	177 (84.7%)		128 (85.3%)	128 (85.3%)	
Corticosteroid application, n (%)			0.009			0.299
Yes	155 (57.4%)	95 (45.5%)		81 (54.0%)	72 (48.0%)	
No	115 (42.6%)	114 (54.5%)		69 (46.0%)	78 (52.0%)	
Osteopenia treatment, n (%)			<0.001			0.590
Yes	63 (25.2%)	24 (11.5%)		16 (10.7%)	19 (12.7%)	
No	207 (76.7%)	185 (88.5%)		134 (89.3%)	131 (87.3%)	

PSM, propensity score matching; BMD, bone mineral density; SD, standard deviation; ICIs, immune checkpoint inhibitors; PD-1, programmed cell death protein 1; PD-L1, programmed cell death ligand 1; COPD, chronic obstructive pulmonary disease; A/G ratio, albumin to globulin ratio; NLR, neutrophil to lymphocyte ratio; PLR, platelet to lymphocyte ratio.

### The optimum cutoff value of BMD

The X-tile software was used to determine the optimal cutoff value for BMD classification (**Supplementary Figures 2A, B**). Specifically, we used the OS outcome as a reference, and on the basis of ensuring that the OS of the two groups of patients were in the same trend at all cutoff points, we selected the point with the most significant difference in the outcomes of the two groups of patients. Finally, the cutoff value was determined to be 138 HU, and 270 patients were classified into the BMD-lower group and 209 patients were classified into the BMD-higher group.

To demonstrate osteopenia, this study performed a correlation analysis between two BMD measurements taken from the first lumbar vertebra and the tenth thoracic vertebra in the same subject. Scatter plots showed significant correlations between the two BMD measurements before (R=0.947, *P*<0.001) and after PSM analysis (R=0.955, *P*<0.001) (**Supplementary Figures 3A, B**).

### Tumor response

The tumor responses of the BMD-lower group and BMD-higher group before and after PSM analysis are shown in **Supplementary Tables 2**, **3**. Overall, short-term therapeutic effects were similar between the two groups. Before PSM analysis, the ORR and DCR of the BMD-lower group were 43.3% and 91.1%, respectively, and were 43.5% and 94.3% in the BMD-higher group, with no statistical difference (ORR, *P* = 0.964; DCR, *P* = 0.195). Similarly, there were also no statistically significant differences in ORR (44.7% vs. 44.7%, *P* ;= 1.000) and DCR (93.3% vs. 96.7%, *P* = 0.190) between the two groups after PSM analysis. It is worth noting that before PSM analysis, compared with the BMD-higher group, the proportion of patients in the BMD-lower group reaching PD was higher (8.9% vs. 5.7%). After PSM analysis, the difference was still existed (6.6% vs. 3.3%).

### SRE

The incidences of SRE in the BMD-lower group and BMD-higher group are shown in **Supplementary Table 4**. Compared with the BMD-higher group, the incidences of SRE in the BMD-lower group was higher (17.4% vs. 4.8%, *P<*0.001).

### Survival analysis

**Figure 2:**
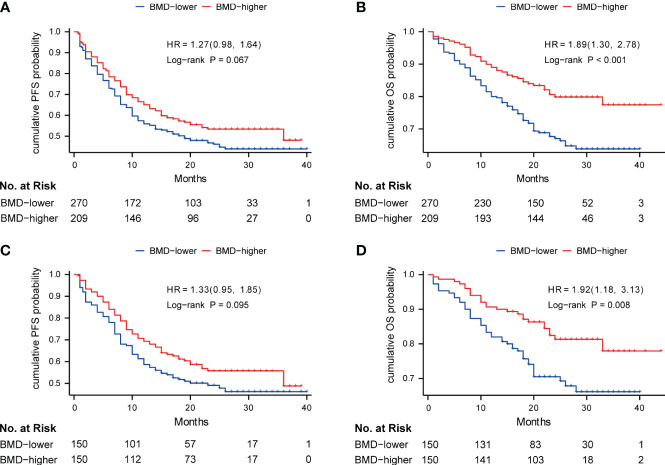
Kaplan-Meier curve of PFS **(A)** and OS **(B)** in the BMD-lower group (blue) and BMD-higher group (red) before PSM analysis; Kaplan-Meier curve of PFS **(C)** and OS **(D)** in two groups after PSM analysis. Analyses were conducted using LogRank tests. BMD, bone mineral density; PFS, progression-free survival; OS, overall survival; PSM, propensity score matching.

The median follow-up time was 22.0 months (IQR, 17.0-29.0 months), and during follow-up, 88 of 270 (32.6%) patients died in the BMD-lower group and 40 of 209 (19.1%) patients died in the BMD-higher group. Kaplan‐Meier survival curves of PFS and OS were conducted between patients with baseline BMD ≤ 138 HU and with baseline BMD > 138 HU. The log-rank tests indicated that the BMD-lower group had the shorter PFS (16.0 months vs. 18.0 months, *P* = 0.067) and OS (20.5 months vs. 23.0 months, *P*< 0.001) than the BMD-higher group before the PSM analysis (**Figures 2A, B**). Likewise, after the PSM analysis, the BMD-lower group still had a shorter PFS (17.0 months vs. 19.0 months, *P* = 0.095) than the BMD-higher group, although there is no statistical difference (**Figure 2C**). And the OS (20.0 months vs. 23.0 months, *P* = 0.008) of the BMD-lower group was significantly shorter than that of the BMD-higher group, reaching statistical significance (**Figure 2D**).

**Figure 3:**
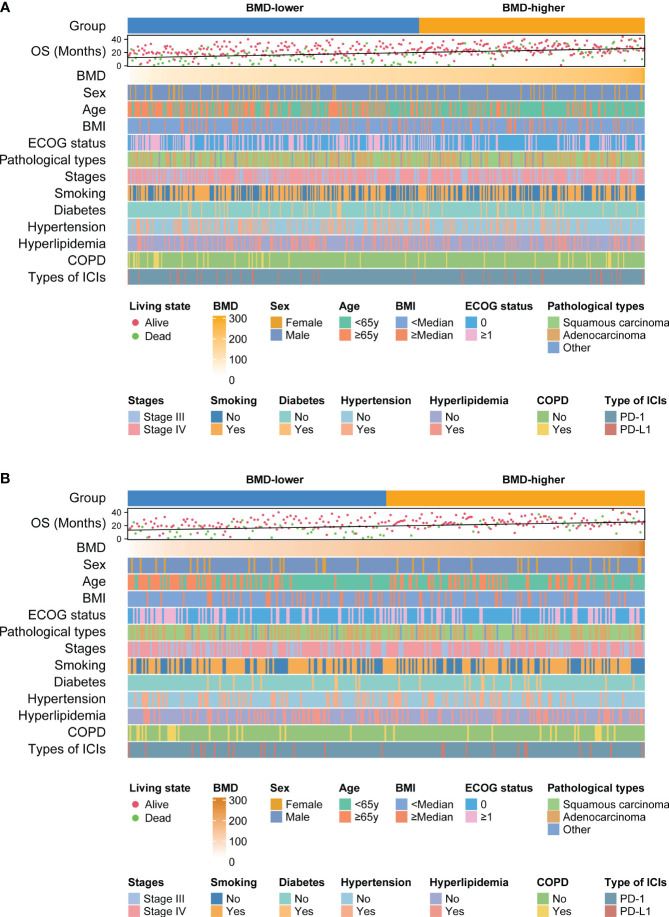
Heat maps shows the relationship between BMD, clinical features of patients and survival outcomes before **(A)** and after **(B)** PSM analysis. BMD, bone mineral density; OS, overall survival; COPD, chronic obstructive pulmonary disease; ICIs, immune checkpoint inhibitors; PD-1, programmed cell death protein 1; PD-L1, programmed cell death ligand 1; PSM, propensity score matching.

Patients were divided into 3 groups according to the tertiles of BMD. Kaplan–Meier curves showed patients in the highest tertile of BMD had better OS compared to those in the lowest tertile before PSM analysis (*P* = 0.030) (**Supplementary Figure 4A**). This trend was still significant after PSM analysis (*P* = 0.042) (**Supplementary Figure 4B**). Meanwhile, we performed a correlation analysis to determine if an association existed between BMD and OS. Scatter plots showed the correlation between BMD and OS before (R=0.325, *P*< 0.001) and after PSM analysis (R=0.337, *P*< 0.001) (**Supplementary Figures 5A, B**). Furthermore, the relationship of BMD, clinical features and survival of patients in different BMD groups before and after PSM analysis are shown in heat maps (**Figures 3A, B**).

To decrease the influence of age on our findings, we calculated each patient’s age-adjusted standard BMD and divided patients into the osteopenia group (n=314) and the non-osteopenia group (n=165) based on the standard BMD. Notably, the OS of the non-osteopenia group was better than that of the osteopenia group (*P* = 0.019) and a similar trend was found in the PFS of the two groups (*P* =0.059) (**Supplementary Figures 6A, B**).

In addition, to explore the predictive value of BMD in non-immunotherapy patients, we randomly selected 100 patients with NSCLC who receive standard chemotherapy at the same time. Patients were divided into the low group (n=65) and the high group (n=35) based on calculated age-adjusted standard BMD values. We found that there were no significant differences in the PFS between the two groups (*P* =0.671) (**Supplementary Figure 7A**). The high group had a longer OS than the low group, although there is no statistical difference (*P* =0.063) (**Supplementary Figure 7B**).

It is worth noting that some patients in the BMD-lower group received osteopenia treatment. After analysis, there were no significant differences in the PFS between the two groups (*P* =0.429) (**Supplementary Figure 8A**). Patients who received osteopenia treatment had a longer OS than those who did not, although the difference did not reach statistical significance (*P* =0.097) (**Supplementary Figure 8B**).

### Cox regression analysis and subgroup analysis

Before PSM analysis, ECOG status, stages, alkaline phosphatase, blood urea nitrogen, albumin to globulin ratio, neutrophil to lymphocyte ratio, platelet to lymphocyte ratio, vertebral bone metastasis, corticosteroid application, skeletal-related events and group were identified as potential predictors for PFS, and age, ECOG status, stages, hypertension, alkaline phosphatase, albumin to globulin ratio, neutrophil to lymphocyte ratio, platelet to lymphocyte ratio, vertebral bone metastasis, corticosteroid application, and group were identified as potential predictors for OS in the univariable regression analysis. These covariates were further included in the multivariate regression analysis. In the multivariate analyses, stage IV (HR, 1.72[95%Cl, 1.21 to 2.46]; *P* =0.003), lower albumin to globulin ratio (HR, 1.75 [95%Cl, 1.12 to 2.70]; *P* = 0.013)and corticosteroid application (HR, 1.39 [95%Cl, 1.06 to 1.81]; *P* = 0.017) were significantly associated with shorter PFS (**Supplementary Table 5**), and aged over 65 years old (HR, 1.53[95%Cl, 1.04 to 2.24]; *P* =0.029), ECOG status ≥1 (HR, 1.43 [95%Cl, 1.00 to 2.05]; *P* = 0.048), stage IV (HR, 1.64[95%Cl, 1.00 to 2.70]; *P* =0.049), higher alkaline phosphatase (HR, 1.00 [95%Cl, 1.00 to 1.01]; *P* = 0.006)and BMD-lower group (HR, 1.60[95%Cl,1.07 to 2.40]; *P* = 0.022) were significantly associated with shorter OS (**Supplementary Table 6**). After PSM analysis, higher albumin to globulin ratio (HR, 1.00 [95%Cl, 1.00 to 1.01]; *P* =0.049) and stage IV (HR, 2.13[95%Cl, 1.35 to 3.33]; *P<*0.001) were significant risk factors associated with a shorter PFS (**Table 2**), and aged over 65 years old (HR, 1.81[95%Cl, 1.10 to 2.97]; *P* = 0.020), stage IV (HR, 1.97 [95%Cl, 1.06 to 3.66]; *P* =0.032), higher alkaline phosphatase (HR, 1.00 [95%Cl, 1.00 to 1.01]; *P* = 0.025), lower albumin to globulin ratio (HR, 2.38 [95%Cl, 1.00 to 5.56]; *P* = 0.044), and BMD-lower group (HR, 1.90 [95%Cl, 1.16 to 3.12]; *P* = 0.011) were significant risk factors associated with a shorter OS (**Table 3**).

**Table 2:** Univariate and multivariate Cox proportional hazards analyses for PFS after PSM analysis.

Parameter	Univariate analysis		Multivariate analysis	
	Hazard ratio (95% CI)	*P* value	Hazard ratio (95% CI)	*P* value
Sex				
Male	Reference			
Female	1.12 (0.68, 1.87)	0.651		
Age				
<65	Reference			
≥65	1.08 (0.76, 1.52)	0.673		
ECOG status				
0	Reference		Reference	
≥1	1.64 (1.17, 2.28)	0.004	1.36 (0.97, 1.91)	0.077
Pathological types				
Squamous carcinoma	Reference			
Adenocarcinoma	1.20 (0.85, 1.69)	0.294		
Other	1.02 (0.51, 2.05)	0.947		
Stages				
Stage III	Reference		Reference	
Stage IV	2.17 (1.54, 3.57)	<0.001	2.13 (1.35, 3.33)	<0.001
Smoking				
No	Reference			
Yes	0.99 (0.71, 1.38)	0.936		
Diabetes				
No	Reference			
Yes	1.23 (0.71, 2.14)	0.464		
Hypertension				
No	Reference			
Yes	1.31 (0.93, 1.85)	0.126		
Hyperlipidemia				
No	Reference			
Yes	0.84 (0.59, 1.21)	0.353		
COPD				
No	Reference			
Yes	1.34 (0.83, 2.18)	0.234		
Alkaline phosphatase	1.00 (1.00, 1.01)	0.018	1.00 (0.99, 1.00)	0.596
Ca	0.50 (0.15, 1.66)	0.260		
Blood urea nitrogen	0.91 (0.83, 1.00)	0.059	0.91 (0.83, 1.00)	0.059
Albumin to globulin ratio	0.55 (0.32, 0.94)	0.028	0.56 (0.32, 1.00)	0.049
Neutrophil to lymphocyte ratio				
≤2	Reference		Reference	
>2	1.47 (0.91, 2.39)	0.116	1.14 (0.68, 1.90)	0.616
Platelet to lymphocyte ratio				
≤150	Reference		Reference	
>150	1.54 (1.08, 2.20)	0.018	1.23 (0.83, 1.82)	0.297
Vertebral bone metastasis				
No	Reference		Reference	
Yes	1.65 (1.09, 2.50)	0.018	1.01 (0.62, 1.65)	0.976
Corticosteroid application				
No	Reference		Reference	
Yes	1.47 (1.05, 2.04)	0.024	1.30 (0.91, 1.84)	0.149
Skeletal-related events				
No	Reference		Reference	
Yes	2.58 (1.51, 4.42)	<0.001	1.83 (1.00, 3.35)	0.051
Group				
BMD- higher	Reference		Reference	
BMD- lower	1.33 (0.95, 1.85)	0.094	1.37 (0.98, 1.92)	0.068

PFS, progression-free survival; PSM, propensity score matching; Cl, confidence interval; COPD, chronic obstructive pulmonary disease; BMD, bone mineral density.

**Table 3:** Univariate and multivariate Cox proportional hazards analyses for OS after PSM analysis.

Parameter	Univariate analysis		Multivariate analysis	
	Hazard ratio (95% CI)	*P* value	Hazard ratio (95% CI)	*P* value
Sex				
Male	Reference			
Female	0.84 (0.39, 1.84)	0.663		
Age				
<65	Reference		Reference	
≥65	1.83 (1.15, 2.92)	0.011	1.81 (1.10, 2.97)	0.020
ECOG status				
0	Reference		Reference	
≥1	1.67 (1.04, 2.66)	0.033	1.33 (0.82, 2.14)	0.251
Pathological types				
Squamous carcinoma	Reference			
Adenocarcinoma	1.03 (0.64, 1.66)	0.897		
Other	0.42 (0.10, 1.74)	0.231		
Stages				
Stage III	Reference		Reference	
Stage IV	2.03 (1.13, 3.65)	0.018	1.97 (1.06, 3.66)	0.032
Smoking				
No	Reference			
Yes	0.92 (0.58, 1.48)	0.744		
Diabetes				
No	Reference			
Yes	1.48 (0.71, 3.10)	0.294		
Hypertension				
No	Reference			
Yes	1.30 (0.80, 2.12)	0.291		
Hyperlipidemia				
No	Reference			
Yes	0.85 (0.51, 1.42)	0.536		
COPD				
No	Reference			
Yes	1.28 (0.65, 2.50)	0.472		
Alkaline phosphatase	1.00 (1.00, 1.01)	0.011	1.00 (1.00, 1.01)	0.025
Ca	0.43 (0.08, 2.36)	0.332		
Blood urea nitrogen	0.95 (0.83, 1.08)	0.410		
Albumin to globulin ratio	0.36 (0.17, 0.78)	0.010	0.42 (0.18, 1.00)	0.044
Neutrophil to lymphocyte ratio				
≤2	Reference			
>2	1.33 (0.68, 2.59)	0.407		
Platelet to lymphocyte ratio				
≤150	Reference			
>150	1.39 (0.84, 2.31)	0.194		
Vertebral bone metastasis				
No	Reference			
Yes	1.51 (0.85, 2.67)	0.157		
Corticosteroid application				
No	Reference		Reference	
Yes	1.72 (1.06, 2.79)	0.027	1.41 (0.86, 2.31)	0.176
Skeletal-related events				
No	Reference		Reference	
Yes	2.49 (1.19, 5.20)	0.015	1.49 (0.69, 3.21)	0.308
Group				
BMD- higher	Reference		Reference	
BMD- lower	1.91 (1.18, 3.10)	0.009	1.90 (1.16, 3.12)	0.011

OS, overall survival; PSM, propensity score matching; Cl, confidence interval; COPD, chronic obstructive pulmonary disease; BMD, bone mineral density.

**Figure 4:**
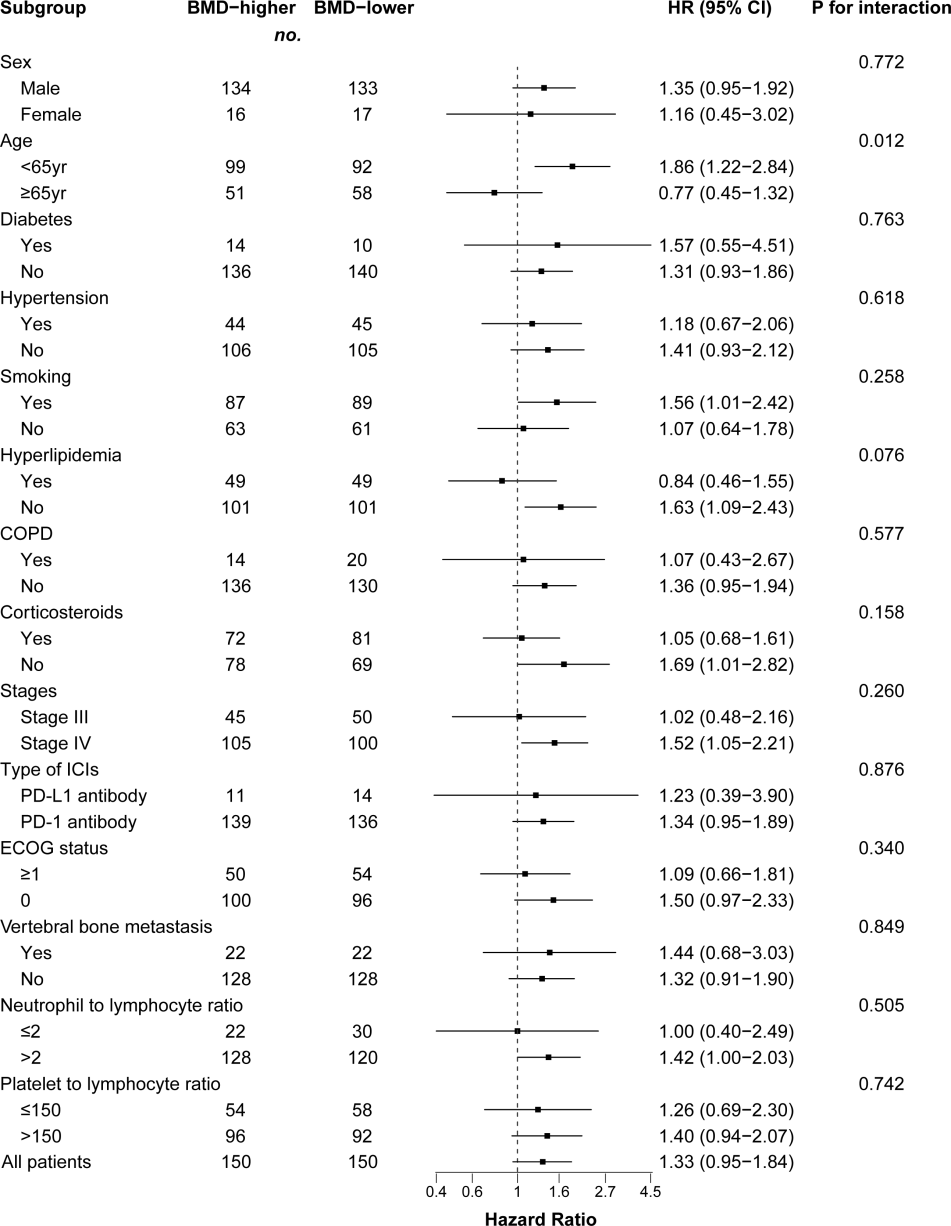
Forest plot of subgroup analysis in progression-free survival between the BMD-lower group and BMD-higher group. Dashed line indicates Hazard ratio of 1. BMD, bone mineral density; HR, hazard ratio; CI, confidence interval; COPD, chronic obstructive pulmonary disease; ICIs, immune checkpoint inhibitors; PD-1, programmed cell death protein 1; PD-L1, programmed cell death ligand 1.

**Figure 5:**
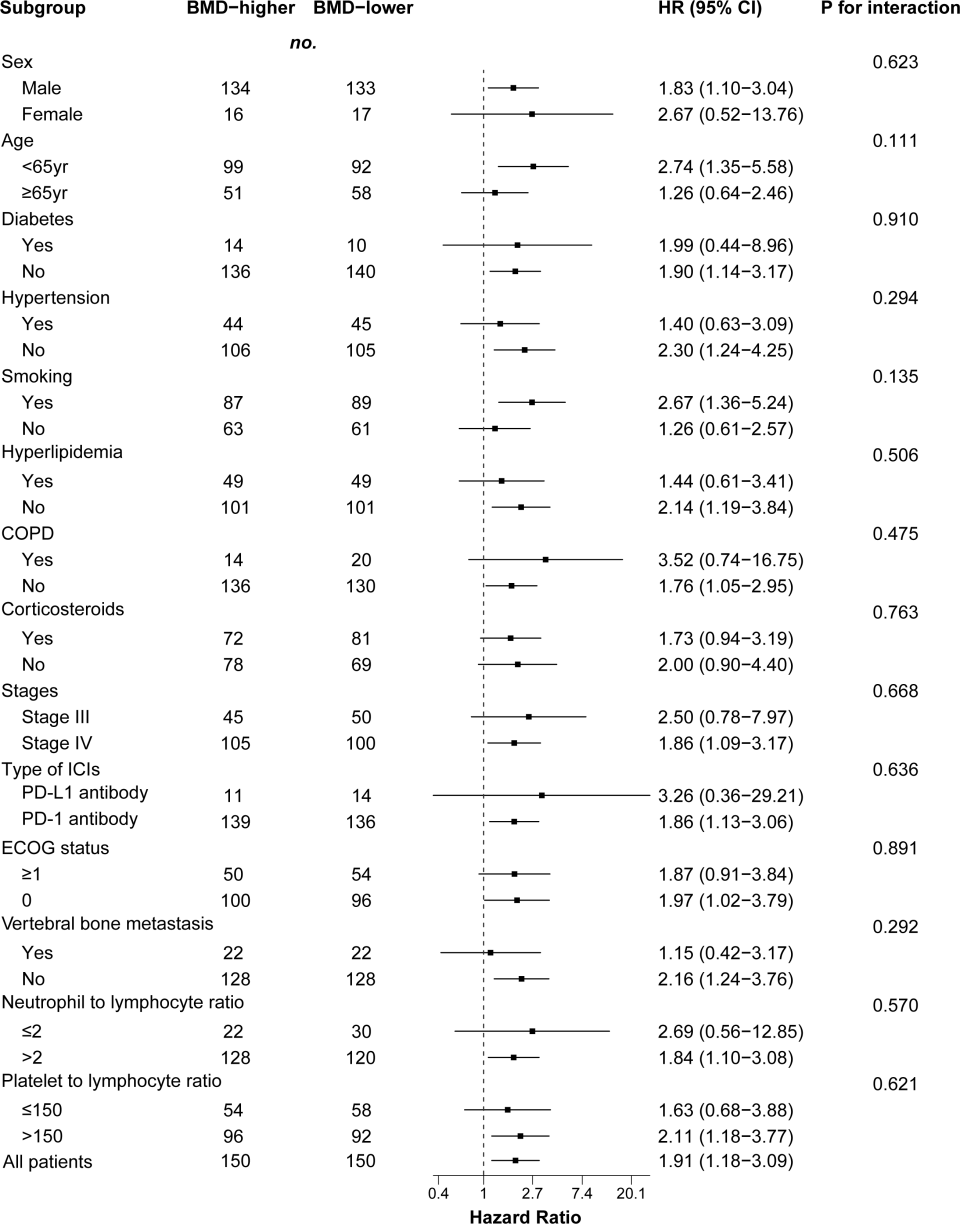
Forest plot of subgroup analysis in overall survival between the BMD-lower group and BMD-higher group. Dashed line indicates Hazard ratio of 1. BMD, bone mineral density; HR, hazard ratio; CI, confidence interval; COPD, chronic obstructive pulmonary disease; ICIs, immune checkpoint inhibitors; PD-1, programmed cell death protein 1; PD-L1, programmed cell death ligand 1.

We performed a subgroup analysis of patients after PSM analysis based on baseline characteristics and observed relatively consistent results for PFS and OS, and hazard ratios for each subgroup were derived from the univariate Cox model. Among each subgroup of PFS (**Figure 4**), we found an interaction between age and ICI use. Except for the subgroup aged ≥ 65 years, the risk of PFS in the BMD-lower group was higher than that in the BMD-higher group. In the subgroup analysis of OS (**Figure 5**), the BMD-lower group showed a higher risk in all subgroups, including those aged ≥ 65 years, although some subgroups did not reach statistical differences.

## Discussion

BMD, as an imaging marker, has been shown to provide potential predictive value for various cancer entities, such as breast cancer and hepatocellular carcinoma (17, 18). This is the first study to report the association between baseline BMD and the short-term efficacy and long-term prognosis in NSCLC patients treated with ICIs. We used X-tile software to scientifically determine the optimal cutoff value. At the same time, considering the impact of factors such as age and sex on BMD and prognosis, we balanced the baseline characteristics of the two groups through PSM, and further analyzed possible influencing factors through Cox regression and subgroup analysis. To reduce the effect of age on study results, we grouped patients again based on the age-adjusted standard BMD. We further explored the predictive value of BMD in non-immunotherapy patients. In this study, CT-derived BMD was used to evaluate baseline BMD. Although DXA was the standard for assessing BMD, more and more studies indicated that CT scans were suitable for predicting vertebral fractures and consecutive measurements of bone loss, correlating well with BMD measured by DXA (19–21). Our results showed that lower baseline BMD was associated with shorter OS, both before and after PSM. Moreover, we found that patients with lower baseline BMD had a higher incidence of SRE. Ilic et al. (22) reported that low preoperative BMD was the independent predictor of patients with NSCLC-related brain metastasis (BM) post-surgical mortality. They measured the BMD value of the first lumbar vertebra in preoperative CT scans and divided it into pathological BMD (median, 99 HU; IQR, 75 to 195 HU) and physiological BMD (median, 140 HU; IQR, 113 to 159 HU) (22). The results showed that pathological BMD was associated with shorter OS (6.0 months vs. 15.0 months, *P* = 0.002) and higher 1-year mortality (OR, 0.5 [95%Cl, 0.2 to 1.0]; *P* = 0.03), which was similar to the results of our study. Similar findings have been reported in other tumors. Watanabe et al. (11) summarized and analyzed 11 studies (2330 patients) on the relationship between gastrointestinal cancer and BMD and found that osteopenia is independently associated with poor prognosis in these patients. The above studies have shown the unique role of BMD in predicting tumor progression. In addition, recent studies and case reports have found that cancer patients have an increased risk of early fractures after starting ICIs, which may be related to reduced bone density and osteoporosis caused by T cell activation (23–25). This suggests that ICIs also promote bone loss and thus affect clinical outcomes, although we did not follow up the changes in BMD after ICI treatment. Due to the double blow of immunotherapy and tumors to bone loss, we should pay more attention to the bone condition of patients before treatment and intervene accordingly.

Our finding suggest that OS was better in patients initially presenting with higher BMD, however there is no difference concerning PFS, ORR or DCR between the two groups. PFS, ORR and DCR have been implemented as early clinical end points and have been extensively used in the evaluation of anti-tumor therapy. However, the relationship between these early end points and OS has not been formally established, which may be influenced by multiple factors (26). Notably, several immunotherapy trials demonstrated improvements in OS without improvements in PFS and/or ORR (27, 28). The ICIs may alter tumor growth kinetics rather than solely act via direct cytotoxicity, which may be the reason for the divorce between ORR, PFS and OS. Furthermore, our study found that corticosteroid application was associated with shorter PFS and was a potential risk factor for OS, although statistical differences were not reached after PSM. Corticosteroids, as immunosuppressive drugs, could exert several mechanisms to reduce immune activity. However, ICIs are designed to enhance the immune system’s inherent antitumor activity (29). Based on our results, we speculate that there may be an antagonistic effect between corticosteroids and ICIs. However, more research is needed to further verify the interactions of corticosteroid use and ICI.

It is still unclear the association between the lower baseline BMD and immunotherapy efficacy and cancer progression, but increasing evidence suggests that the immune systems are closely closed to skeletal systems. Cytokines (such as PTHrP, interleukin (IL)-1, IL-6, and IL-8) derived from cancer cells could activate osteoclasts and subsequently activate the RANKL/RANK pathway which had been proven to be correlated with poor prognosis in cancer patients (30, 31). Animal experiments have shown that reduced bone mineral density, trabecular thickness, and mineralization can be observed in NSCLC mice in the absence of tumor cell metastasis (32). From another point of view, BMD not only reflected the general condition and nutritional status of patients but was also associated with tumor progression to a degree. Bisphosphonates and Denosumab, the widely-used long-term treatment of osteolytic bone diseases and bone metastasis, were reported to exert direct and indirect anti-tumor effects, including inhibition of tumor cell proliferation and adhesion, reduction tumor cells secrete factors that increase RANKL expression, enhancement of immune surveillance, and prevention of angiogenesis (33–35). Clinical and preclinical experiments have shown that they can not only inhibit the progression of bone metastases and reduce SREs, but also prevent the growth of non-small cell lung cancer (36–38).

Our study had limitations. First, this study was retrospective, and data was collected previously. Prospective multicenter clinical trials are needed to validate our results in the future. Second, it should be considered whether the evaluation of CT-derived BMD was reliable, although there have been a number of previous studies using this method. Finally, bone loss was a continuous pathological process under the long-lasting negative effects of tumor, but we did not evaluate changes in BMD during follow-up. Therefore, besides the baseline BMD, the extent of decline in BMD during ICI treatment and its association with prognosis should also be investigated with great care in the future studies. In spite of these limitations, we reported the relationship between baseline BMD and prognosis in NSCLC patients treated with ICIs for the first time.

## Conclusions

In conclusion, our study found that for patients with NSCLC, baseline BMD before ICI treatment affects the long-term prognosis of them, although there is no difference in their short-term efficacy. Routine testing of BMD before receiving immunotherapy will help clinicians make better decisions.

## Data availability statement

The raw data supporting the conclusions of this article will be made available by the authors, without undue reservation.

## Ethics statement

The studies involving humans were approved by The Research Ethics Committee of Tongji Medical College, Huazhong University of Science and Technology (Institutional Review Board No. S054). The studies were conducted in accordance with the local legislation and institutional requirements. The ethics committee/institutional review board waived the requirement of written informed consent for participation from the participants or the participants’ legal guardians/next of kin because clinical data were analyzed retrospectively and anonymously.

## Author contributions

JL: Conceptualization, Data curation, Writing – original draft. BG: Data curation, Writing – original draft. YL: Data curation, Writing – original draft. YG: Conceptualization, Data curation, Resources, Writing – original draft. LL: Data curation, Writing – original draft. JW: Investigation, Project administration, Supervision, Writing – review & editing. WL: Data curation, Resources, Validation, Writing – original draft. ZY: Data curation, Resources, Writing – original draft. HZ: Investigation, Methodology, Writing – original draft. FP: Software, Validation, Writing – review & editing. BL: Formal Analysis, Project administration, Supervision, Writing – review & editing. LY: Project administration, Supervision, Visualization, Writing – review & editing. GZ: Conceptualization, Project administration, Supervision, Writing – review & editing.
