# Supplementary material for: Bone mineral density as an individual prognostic biomarker in NSCLC patients treated with immune checkpoint inhibitors
Source: Front Immunol. 2024 Apr 18;15:1332303. doi: 10.3389/fimmu.2024.1332303 (PMC11063287; doi:10.3389/fimmu.2024.1332303)

**SUPPLEMENTAL MATERIAL**

**Supplementary Table 1. Baseline Characteristics of Patients Excluded by PSM**

| Characteristics | Patients Excluded by PSM (n=179) |
| --- | --- |
| Sex, n (%) |  |
| Male | 153 (85.5%) |
| Female | 26 (14.5%) |
| Age, n (%) |  |
| <65 | 99 (55.3%) |
| ≥65 | 80 (44.7%) |
| Body mass index, n (%) |  |
| <Median | 150 (83.8%) |
| ≥Median | 29 (16.2%) |
| ECOG status, n (%) |  |
| 0 | 104 (58.1%) |
| ≥1 | 75 (41.9%) |
| Pathological types, n (%) |  |
| Squamous carcinoma | 75 (41.9%) |
| Adenocarcinoma | 93 (52.0%) |
| Other | 11 (6.1%) |
| Stages, n (%) |  |
| Stage III | 39 (21.8%) |
| Stage IV | 140 (78.2%) |
| Type of ICIs, n (%) |  |
| PD-1 | 168 (93.9%) |
| PD-L1 | 11 (6.1%) |
| Smoking, n (%) |  |
| Yes | 84 (46.9%) |
| No | 95 (53.1%) |
| Diabetes, n (%) |  |
| Yes | 21 (11.7%) |
| No | 158 (88.3%) |
| Hypertension, n (%) |  |
| Yes | 62 (34.6%) |
| No | 117 (65.4%) |
| Hyperlipidemia, n (%) |  |
| Yes | 51 (28.5%) |
| No | 128 (71.5%) |
| COPD, n (%) |  |
| Yes | 11 (6.1%) |
| No | 168 (93.9%) |
| Alkaline phosphatase, mean (SD) | 92 (72, 118) |
| Lactic dehydrogenase, mean (SD) | 213 (178.5, 271.5) |
| Ca, mean (SD) | 2.2 (2.1, 2.3) |
| Blood urea nitrogen, mean (SD) | 5.2 (4.0, 6.2) |
| Hemoglobin, mean (SD) |  |
| A/G ratio, mean (SD) | 1.4 (1.2, 1.6) |
| NLR, n (%) |  |
| ≤2 | 26 (14.5%) |
| >2 | 153 (85.5%) |
| PLR, n (%) |  |
| ≤150 | 60 (33.5%) |
| >150 | 119 (66.5%) |
| Prior radiation therapy, n (%) |  |
| Yes | 27 (15.1%) |
| No | 152 (84.9%) |
| Vertebral bone metastasis, n (%) |  |
| Yes | 56 (31.3%) |
| No | 123 (68.7%) |
| Corticosteroid application, n (%) |  |
| Yes | 97 (54.2%) |
| No | 82 (45.8%) |
| Osteopenia treatment, n (%) |  |
| Yes | 52 (29.1%) |
| No | 127 (70.9%) |

Abbreviations: PSM, propensity score matching; BMD, bone mineral density; SD, standard deviation; ICIs, immune checkpoint inhibitors; PD-1, programmed cell death protein 1; PD-L1, programmed cell death ligand 1; COPD, chronic obstructive pulmonary disease; A/G ratio, albumin to globulin ratio; NLR, neutrophil to lymphocyte ratio; PLR, platelet to lymphocyte ratio.

**Supplementary Table 2. Tumor Response Between the BMD-lower Group and BMD-higher Group Before PSM Analysis**

| Tumor response | BMD-lower (n=270) | BMD-higher (n=209) | *P* value |
| --- | --- | --- | --- |
| CR, n (%) | 0 (0.0%) | 0 (0.0%) |  |
| PR, n (%) | 117 (43.3%) | 91 (43.3%) |  |
| SD, n (%) | 129 (47.8%) | 106 (50.7%) |  |
| PD, n (%) | 24 (8.9%) | 12 (5.7%) |  |
| ORR% | 43.3% | 43.5% | 0.964 |
| DCR% | 91.1% | 94.3% | 0.195 |

Abbreviations: BMD, bone mineral density; PSM, propensity score matching; CR, complete response; PR, partial response; SD, stable disease; PD, progressive disease; ORR, objective response rate; DCR, disease control rate.

**Supplementary Table 3. Tumor Response Between the BMD-lower Group and BMD-higher Group after PSM Analysis**

| Tumor response | BMD-lower (n=150) | BMD-higher (n=150) | *P* value |
| --- | --- | --- | --- |
| CR, n (%) | 0 (0.0%) | 0 (0.0%) |  |
| PR, n (%) | 67 (44.7%) | 67 (44.7%) |  |
| SD, n (%) | 73 (48.7%) | 78 (52.0%) |  |
| PD, n (%) | 10 (6.6%) | 5 (3.3%) |  |
| ORR% | 44.7% | 44.7% | 1.000 |
| DCR% | 93.3% | 96.7% | 0.190 |

Abbreviations: BMD, bone mineral density; PSM, propensity score matching; CR, complete response; PR, partial response; SD, stable disease; PD, progressive disease; ORR, objective response rate; DCR, disease control rate.

**Supplementary Table 4. Incidence of skeletal-related events between the BMD-lower Group and BMD-higher Group.**

|  | BMD-lower(n=270) | BMD-higher(n=209) | *P* value |
| --- | --- | --- | --- |
| SRE, n(%) | 47(17.4%) | 10(4.8%) | <0.001 |

Abbreviations: BMD, bone mineral density; SRE, skeletal-related events.

**Supplementary Table 5.** **Cox Proportional Hazards Analyses for PFS Before PSM** **Analysis**

| Parameter | Univariate analysis | | Multivariate analysis | |
| --- | --- | --- | --- | --- |
|  | Hazard ratio (95% CI) | *P* value | Hazard ratio (95% CI) | *P* value |
| Gender |  |  |  |  |
| Male | Reference |  |  |  |
| Female | 1.09 (0.75, 1.59) | 0.645 |  |  |
| Age |  |  |  |  |
| <65 | Reference |  |  |  |
| ≥65 | 1.10 (0.85, 1.42) | 0.464 |  |  |
| ECOG status |  |  |  |  |
| 0 | Reference |  | Reference |  |
| ≥1 | 1.46 (1.13, 1.89) | 0.003 | 1.28 (0.98, 1.66) | 0.067 |
| Pathological types |  |  |  |  |
| Squamous carcinoma | Reference |  |  |  |
| Adenocarcinoma | 1.27 (0.98, 1.65) | 0.068 |  |  |
| Other | 0.86 (0.48, 1.52) | 0.596 |  |  |
| Stages |  |  |  |  |
| Stage III | Reference |  | Reference |  |
| Stage IV | 2.07 (1.50, 2.87) | <0.001 | 1.72 (1.21, 2.46) | 0.003 |
| Smoking |  |  |  |  |
| No | Reference |  |  |  |
| Yes | 0.83 (0.65, 1.07) | 0.160 |  |  |
| Diabetes |  |  |  |  |
| No | Reference |  |  |  |
| Yes | 1.39 (0.93, 2.07) | 0.108 |  |  |
| Hypertension |  |  |  |  |
| No | Reference |  |  |  |
| Yes | 1.21 (0.93, 1.57) | 0.163 |  |  |
| Hyperlipidemia |  |  |  |  |
| No | Reference |  |  |  |
| Yes | 0.95 (0.72, 1.25) | 0.718 |  |  |
| COPD |  |  |  |  |
| No | Reference |  |  |  |
| Yes | 1.26 (0.84, 1.89) | 0.275 |  |  |
| Alkaline phosphatase | 1.00 (1.00, 1.01) | <0.001 | 1.00 (1.00, 1.01) | 0.139 |
| Ca | 0.46 (0.18, 1.17) | 0.102 |  |  |
| Blood urea nitrogen | 0.94 (0.87, 1.01) | 0.097 | 0.94 (0.87, 1.01) | 0.089 |
| Albumin to globulin ratio | 0.58 (0.38, 0.88) | 0.010 | 0.57 (0.37, 0.89) | 0.013 |
| Neutrophil to lymphocyte ratio |  |  |  |  |
| ≤2 | Reference |  | Reference |  |
| >2 | 1.59 (1.08, 2.34) | 0.020 | 1.19 (0.78, 1.80) | 0.420 |
| Platelet to lymphocyte ratio |  |  |  |  |
| ≤150 | Reference |  | Reference |  |
| >150 | 1.52 (1.15, 2.00) | 0.003 | 1.24 (0.91, 1.68) | 0.170 |
| Vertebral bone metastasis |  |  |  |  |
| No | Reference |  | Reference |  |
| Yes | 1.70 (1.27, 2.25) | <0.001 | 1.38 (0.98, 1.94) | 0.062 |
| Corticosteroid application |  |  |  |  |
| No | Reference |  | Reference |  |
| Yes | 1.51 (1.17, 1.95) | 0.002 | 1.39 (1.06, 1.81) | 0.017 |
| Skeletal-related events |  |  |  |  |
| No | Reference |  | Reference |  |
| Yes | 1.48 (1.04, 2.10) | 0.029 | 0.82 (0.54, 1.24) | 0.345 |
| Group |  |  |  |  |
| BMD- higher | Reference |  | Reference |  |
| BMD- lower | 1.27 (0.98, 1.65) | 0.067 | 1.25 (0.95, 1.64) | 0.113 |

Abbreviations: PFS, progression-free survival; PSM, propensity score matching; Cl, confidence interval; PD-1, programmed cell death protein 1; PD-L1, programmed cell death ligand 1; BMD, bone mineral density.

**Supplementary Table 6.** **Cox Proportional Hazards Analyses for OS Before PSM** **Analysis**

| Parameter | Univariate analysis | | Multivariate analysis | |
| --- | --- | --- | --- | --- |
|  | Hazard ratio (95% CI) | *P* value | Hazard ratio (95% CI) | *P* value |
| Gender |  |  |  |  |
| Male | Reference |  |  |  |
| Female | 0.86 (0.50, 1.50) | 0.600 |  |  |
| Age |  |  |  |  |
| <65 | Reference |  | Reference |  |
| ≥65 | 1.92 (1.36, 2.72) | **<0.001** | 1.53 (1.04, 2.24) | **0.029** |
| ECOG status |  |  |  |  |
| 0 | Reference |  | Reference |  |
| ≥1 | 1.81 (1.28, 2.56) | **<0.001** | 1.43 (1.00, 2.05) | **0.048** |
| Pathological types |  |  |  |  |
| Squamous carcinoma | Reference |  |  |  |
| Adenocarcinoma | 1.20 (0.84, 1.71) | 0.319 |  |  |
| Other | 0.64 (0.26, 1.61) | 0.345 |  |  |
| Stages |  |  |  |  |
| Stage III | Reference |  | Reference |  |
| Stage IV | 1.92 (1.22, 3.02) | **0.005** | 1.64 (1.00, 2.70) | **0.049** |
| Smoking |  |  |  |  |
| No | Reference |  |  |  |
| Yes | 0.97 (0.69, 1.37) | 0.864 |  |  |
| Diabetes |  |  |  |  |
| No | Reference |  |  |  |
| Yes | 1.52 (0.90, 2.57) | 0.117 |  |  |
| Hypertension |  |  |  |  |
| No | Reference |  | Reference |  |
| Yes | 1.66 (1.17, 2.35) | **0.005** | 1.38 (0.96, 1.99) | 0.082 |
| Hyperlipidemia |  |  |  |  |
| No | Reference |  |  |  |
| Yes | 1.09 (0.75, 1.57) | 0.663 |  |  |
| COPD |  |  |  |  |
| No | Reference |  |  |  |
| Yes | 1.20 (0.69, 2.09) | 0.526 |  |  |
| Alkaline phosphatase | 1.00 (1.00, 1.01) | **0.002** | 1.00 (1.00, 1.01) | **0.006** |
| Ca | 0.39 (0.11, 1.37) | 0.141 |  |  |
| Blood urea nitrogen | 0.97 (0.87, 1.07) | 0.506 |  |  |
| Albumin to globulin ratio | 0.48 (0.27, 0.87) | **0.015** | 0.55 (0.29, 1.05) | 0.068 |
| Neutrophil to lymphocyte ratio |  |  |  |  |
| ≤2 | Reference |  | Reference |  |
| >2 | 1.74 (1.00, 2.59) | 0.051 | 1.19 (0.66, 2.16) | 0.559 |
| Platelet to lymphocyte ratio |  |  |  |  |
| ≤150 | Reference |  | Reference |  |
| >150 | 1.45 (0.99, 2.12) | 0.058 | 1.21 (0.80, 1.82) | 0.368 |
| Vertebral bone metastasis |  |  |  |  |
| No | Reference |  | Reference |  |
| Yes | 1.43 (0.97, 2.12) | 0.069 | 1.04 (0.68, 1.57) | 0.868 |
| Corticosteroid application |  |  |  |  |
| No | Reference |  | Reference |  |
| Yes | 1.56 (1.09, 2.22) | **0.015** | 1.36 (0.94, 1.98) | 0.102 |
| Skeletal-related events |  |  |  |  |
| No | Reference |  |  |  |
| Yes | 1.30 (0.80, 2.12) | 0.288 |  |  |
| Group |  |  |  |  |
| BMD- higher | Reference |  | Reference |  |
| BMD- lower | 1.89 (1.30, 2.75) | **<0.001** | 1.60 (1.07, 2.40) | **0.022** |

Abbreviations: OS, overall survival; PSM, propensity score matching; Cl, confidence interval; PD-1, programmed cell death protein 1; PD-L1, programmed cell death ligand 1; BMD, bone mineral density.


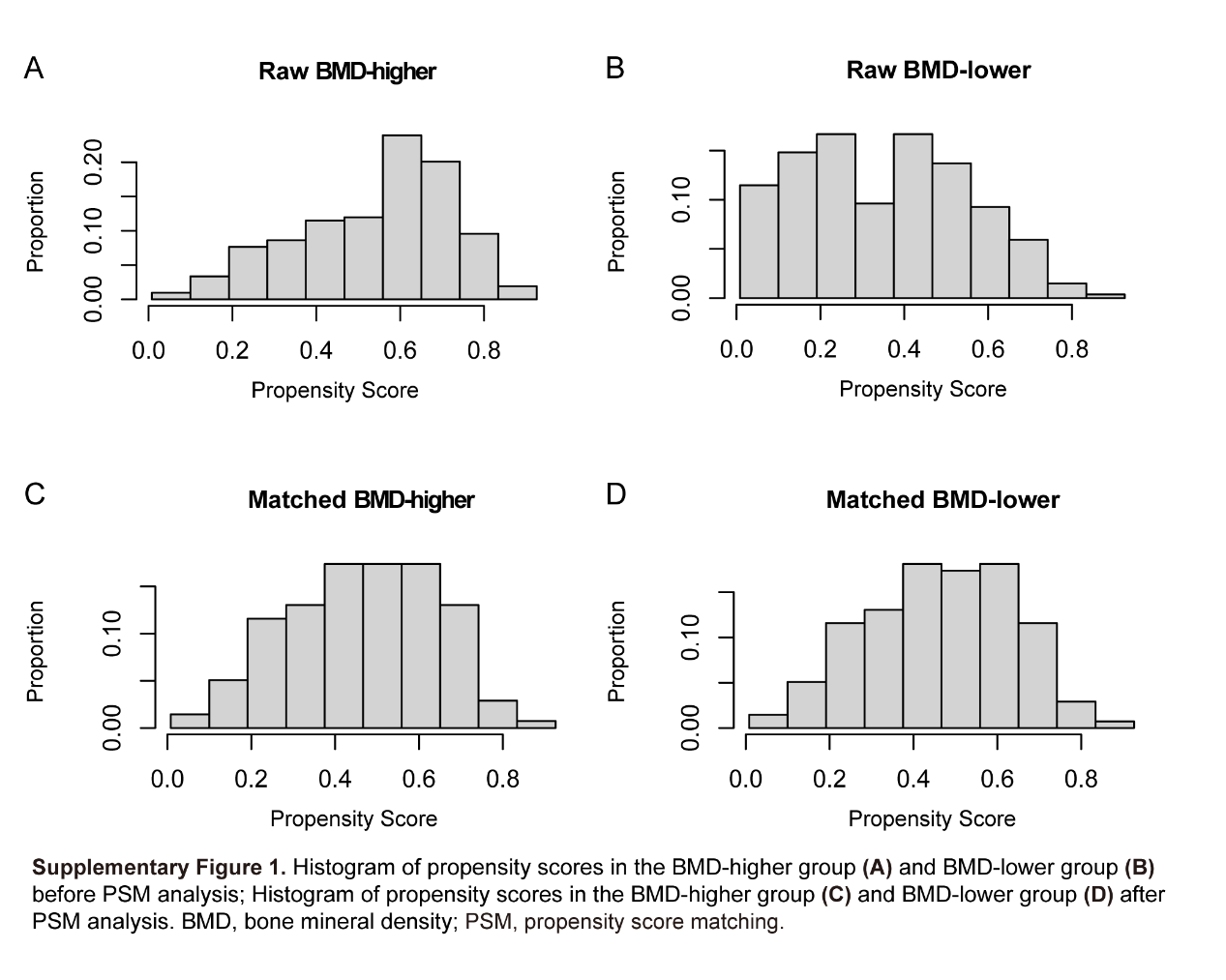


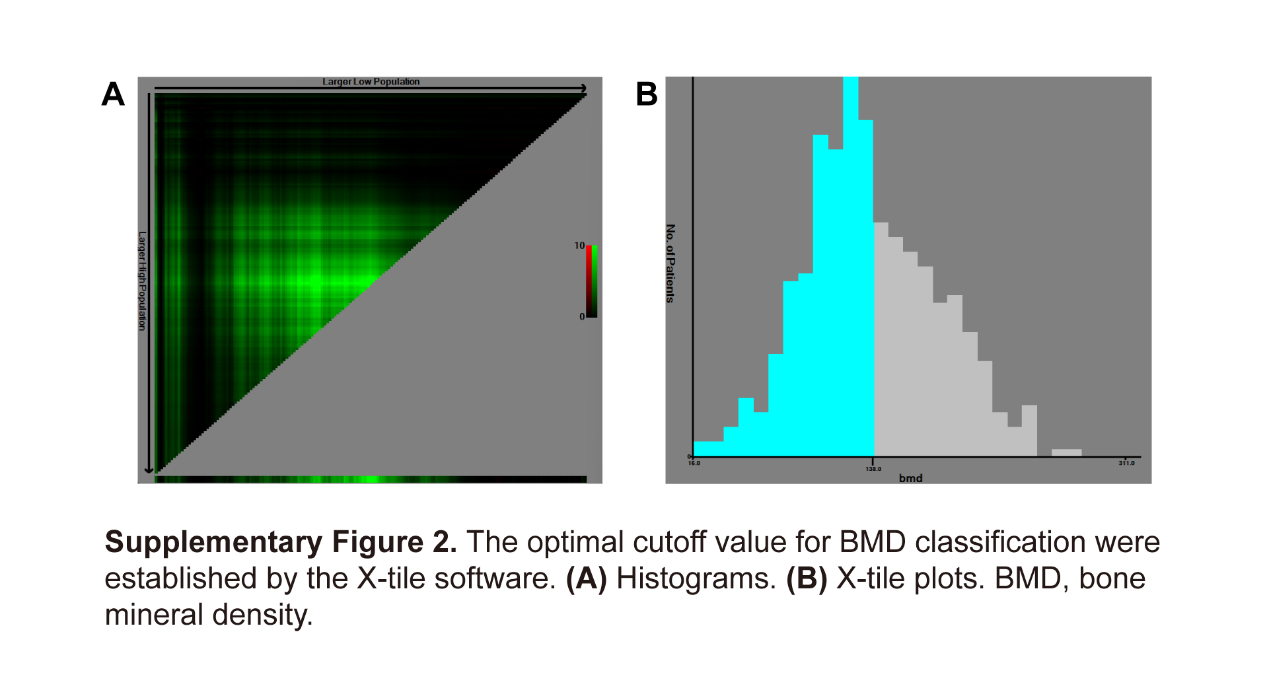


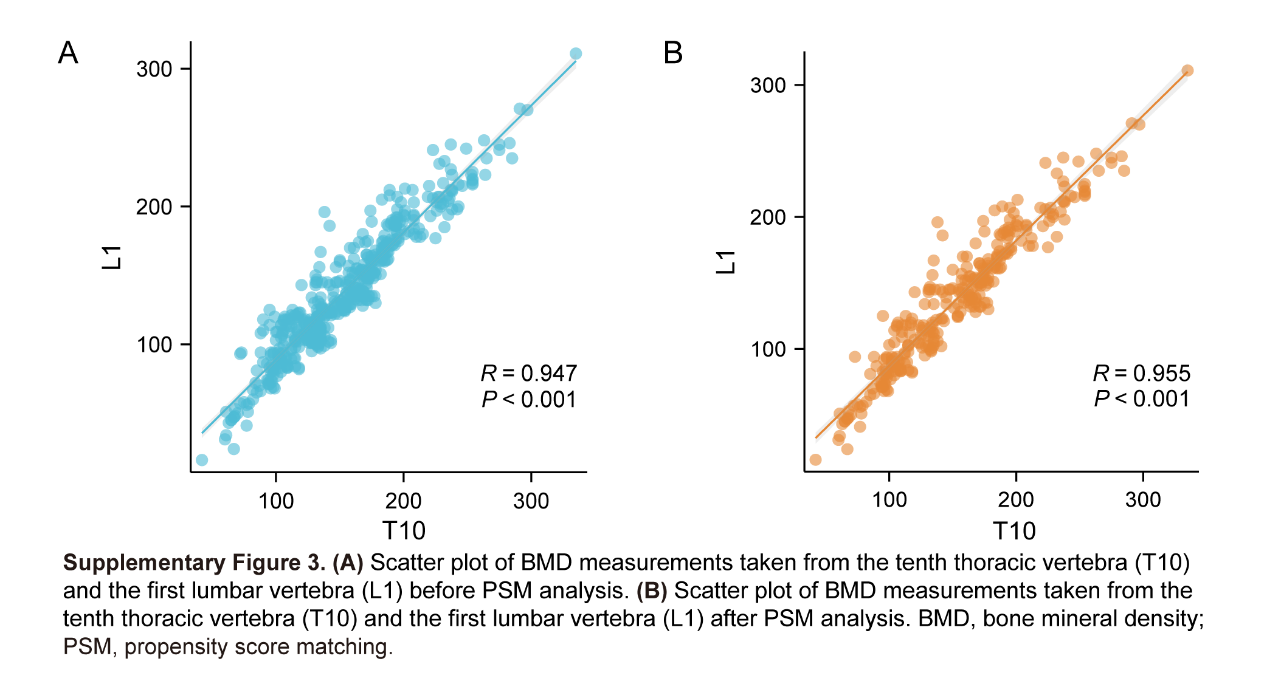


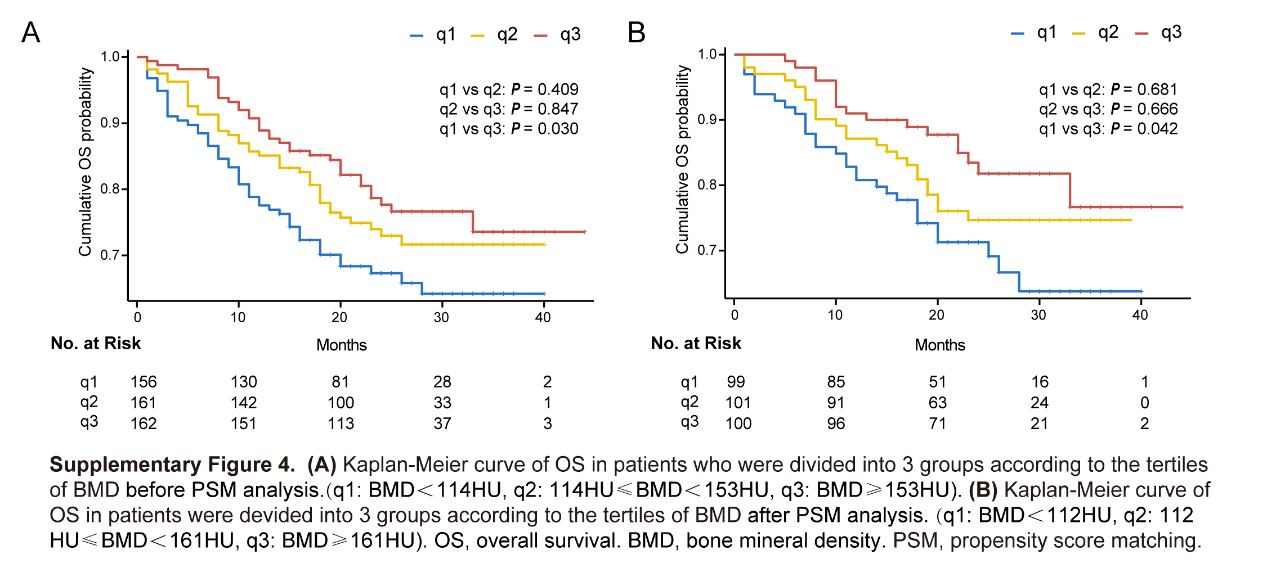


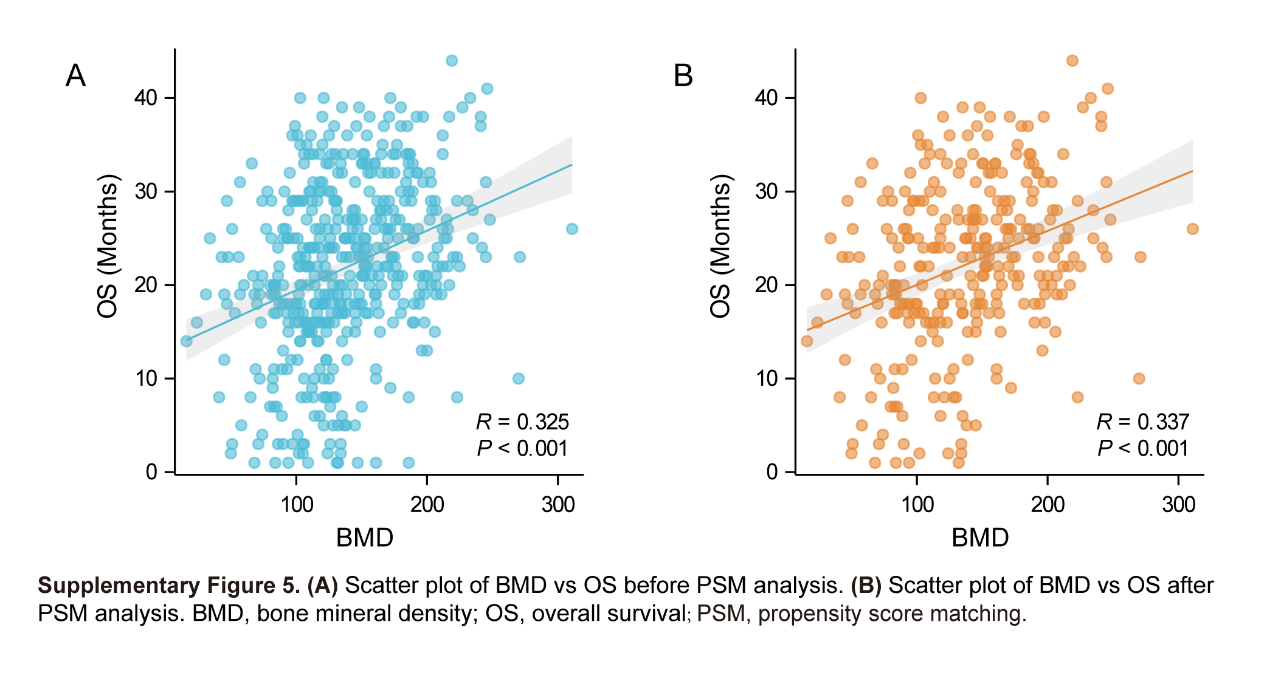


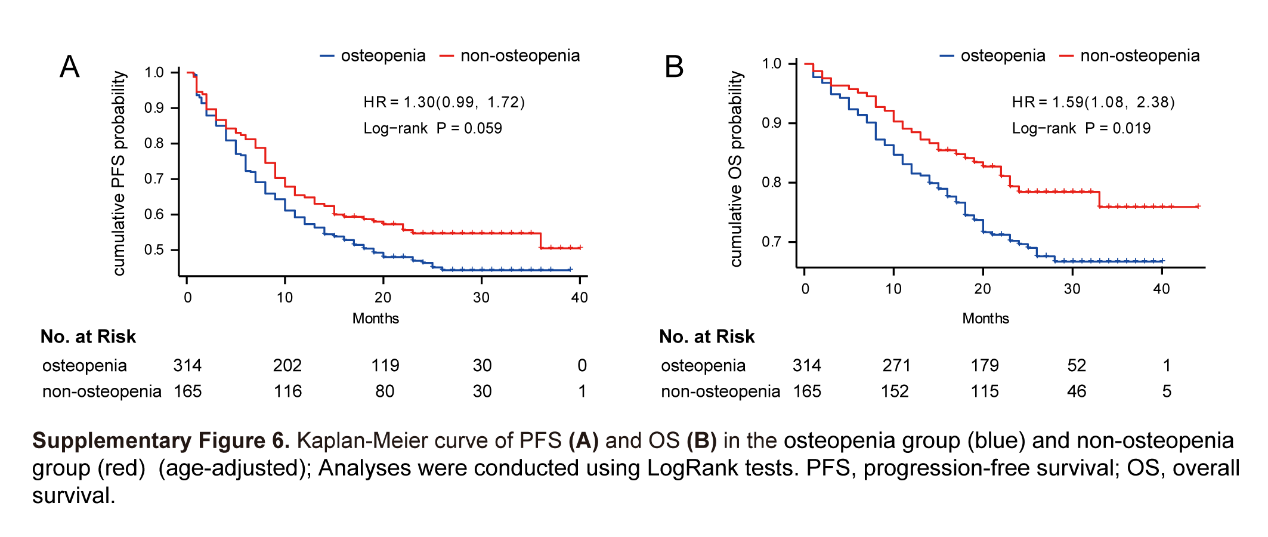


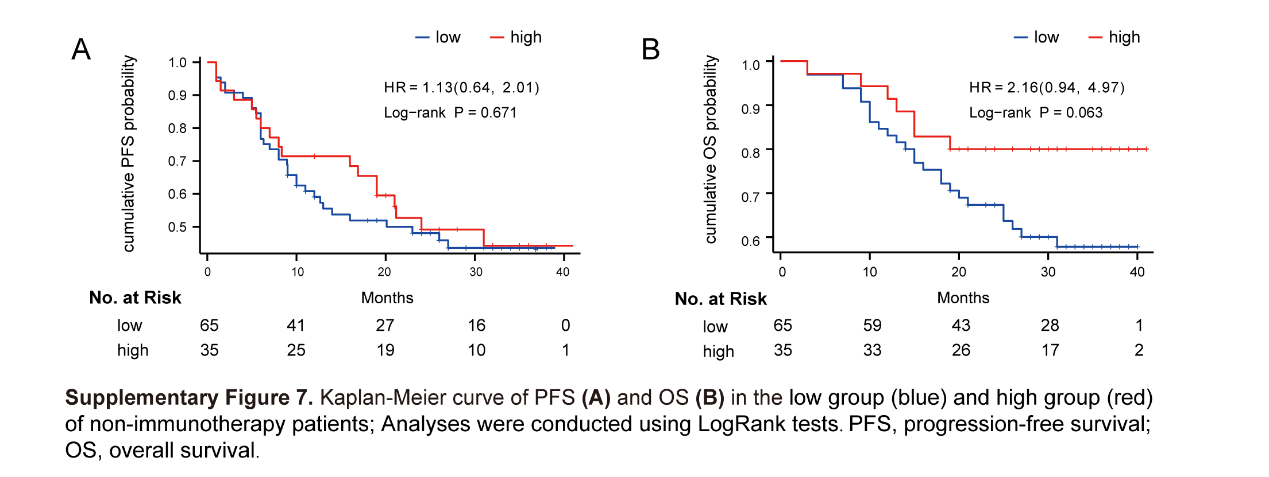


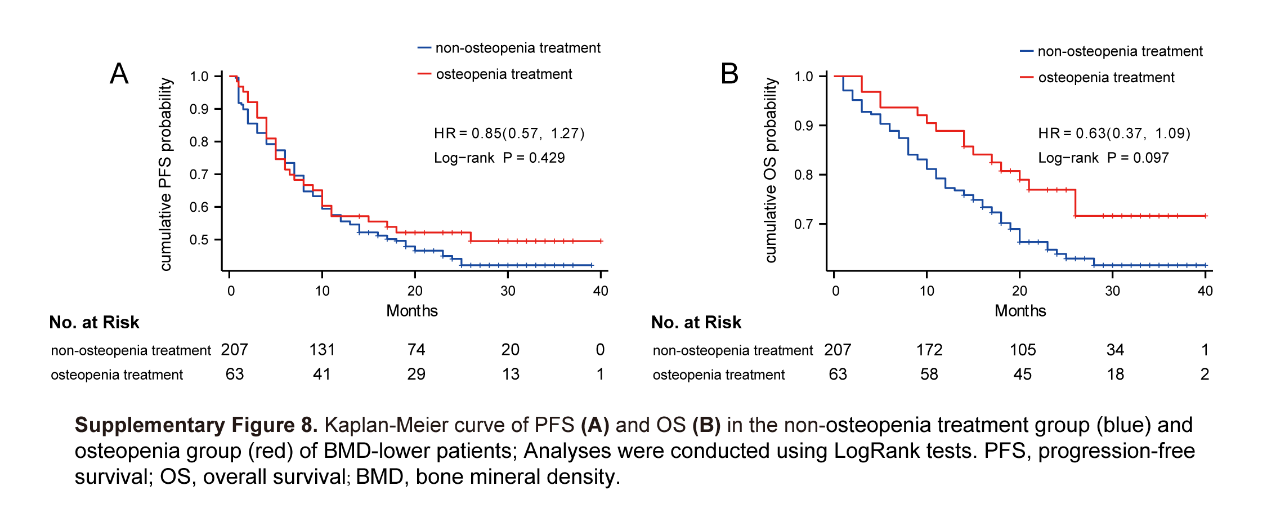

Supplement: Supplementary file 1 [file DataSheet_1.docx]
